# Exosomes and breast cancer drug resistance

**DOI:** 10.1038/s41419-020-03189-z

**Published:** 2020-11-17

**Authors:** Xingli Dong, Xupeng Bai, Jie Ni, Hao Zhang, Wei Duan, Peter Graham, Yong Li

**Affiliations:** 1 Department of Biopharmaceutical Sciences, College of Pharmacy, Harbin Medical University, 150081 Harbin, Heilongjiang China; 2 St George and Sutherland Clinical School, Faculty of Medicine, UNSW Sydney, Kensington, NSW 2052 Australia; 3 Cancer Care Centre, St. George Hospital, Kogarah, NSW 2217 Australia; 4 Institute of Precision Cancer Medicine and Pathology and Department of Pathology, Jinan University Medical College, 510630 Guangzhou, China; 5 School of Medicine and Centre for Molecular and Medical Research, Deakin University, Waurn Ponds, VIC 3216 Australia; 6 School of Basic Medicine Sciences, Zhengzhou University, 450001 Henan, China

**Keywords:** Cancer therapeutic resistance, Medical research

## Abstract

Drug resistance is a daunting challenge in the treatment of breast cancer (BC). Exosomes, as intercellular communicative vectors in the tumor microenvironment, play an important role in BC progression. With the in-depth understanding of tumor heterogeneity, an emerging role of exosomes in drug resistance has attracted extensive attention. The functional proteins or non-coding RNAs contained in exosomes secreted from tumor and stromal cells mediate drug resistance by regulating drug efflux and metabolism, pro-survival signaling, epithelial–mesenchymal transition, stem-like property, and tumor microenvironmental remodeling. In this review, we summarize the underlying associations between exosomes and drug resistance of BC and discuss the unique biogenesis of exosomes, the change of exosome cargo, and the pattern of release by BC cells in response to drug treatment. Moreover, we propose exosome as a candidate biomarker in predicting and monitoring the therapeutic drug response of BC and as a potential target or carrier to reverse the drug resistance of BC.

## Facts

- Tumor-derived exosomes mediate the chemoresistance of BC by reducing the intracellular accumulation of chemotherapeutic drugs and delivering functional cargos that activate pro-survival signaling and unchecked cell cycle progression.
- Tumor-derived exosomes mediate the hormonal resistance of BC by transferring functional cargos that upregulate ERα expression and hormone-independent signaling.
- Tumor-derived exosomes mediate HER2-targeted drug resistance of BC by direct interaction of HER2-overexpressing exosomes with targeted drugs or by transcriptome reprogramming of the HER2^+^ BC into a HER2-independent pattern.
- Tumor- and TME-derived exosomes carrying immunosuppressive molecules mediate the immunotherapeutic resistance of BC by remodeling the tumor immune microenvironment.
- Tumor-derived exosomes mediate enhanced EMT and stem-like property of drug-resistant BC.
- TME-derived exosomes mediate the tumor microenvironmental remodeling that favors the drug resistance of BC.
- The exosome is proposed as a candidate biomarker in predicting and monitoring the therapeutic drug response of BC.
- The exosome is proposed as a potential therapeutic target or carrier to reverse drug resistance.

## Open questions

- How do exosomes mediate drug resistance in BC?
- By what mechanisms do exosomes promote BC progression?
- What is the role of exosomes in mediating the phenotypic transformation of BC, such as enhanced stemness and EMT?
- How do exosomes induce tumor microenvironmental remodeling to facilitate BC drug resistance?
- Do exosomes correlate with immunotherapeutic resistance in BC?
- How can exosomes be utilized as a biomarker to predict and monitor the therapeutic drug response in BC patients?
- What are the necessary steps that will lead to the clinical applicability of the exosomes as biomarkers?
- How can exosomes be applied as a target to overcome drug resistance in BC?
- How can exosomes be applied as a drug carrier in the context of drug resistance in BC?

## Introduction

Breast cancer (BC) is the most commonly diagnosed cancer and the leading cause of cancer-related death in females worldwide^1^. According to the Globocan statistics, there were about 2.1 million newly diagnosed female BC cases in 2018, accounting for approximately 25% of cancer cases in women. Clinically, conventional drugs, such as chemotherapeutic, hormonal, and targeted drugs^2^, remain the first-line drug treatment. Several immunotherapeutic drugs demonstrated promising efficacy in clinical trials^3^. Nevertheless, the treatment outcome is often restricted by drug resistance, as well as the lack of biomarkers for predicting the treatment response. Therefore, further understanding the potential molecular mechanisms responsible for drug resistance and seeking reliable biomarkers to predict and monitor the therapeutic response are necessary for a more effective BC treatment.

Over the past decades, mounting studies demonstrated that the tumor microenvironment (TME) is a major determinant not only of tumor growth, progression, and metastasis but also of treatment resistance^4–8^. Extracellular vesicles (EVs) are heterogeneous populations of small membrane vesicles that are released by cells, carrying a variety of functional nucleic acids, proteins, and lipid cargos important in cell–cell communication^9–12^. According to the size and source, EVs are classified into exosomes (50–100 nm in diameter; endosomal origin)^13^, ectosomes (100–1000 nm in diameter; direct budding of the plasma membrane, also known as microparticles/microvesicles)^14^, apoptotic bodies (1–5 μm in diameter)^15^, large oncosomes (1–10 μm)^9^, and other miscellaneous EV subsets^13^. Exosomes are of particular interest in biological research because their unique biogenesis involves distinct intracellular regulatory processes that give them specific cargos and thus different biological functions^14–16^. Exosomes transfer these cargos to recipient cells and induce their phenotypic changes. Increasing evidence indicates that exosome-mediated cell communication is a newly identified mechanism underlying drug resistance^11,17^. By directly exporting drugs, inducing drug inactivation, and delivering functional proteins and non-coding RNAs, exosomes contribute significantly to BC drug resistance^18,19^.

BC is a highly heterogeneous disease comprised of multiple distinct subtypes, which are different in genetics, pathology, and clinic behaviour, while exosomes mediate drug resistance through different mechanisms in different subtypes of BC, suggesting an extensive role of exosomes in BC progression. In this review, we summarize the latest progress on the association of exosomal cargos with BC drug resistance (Table 1). We also discuss the potential role of exosomes as cancer biomarkers to reflect TME changes and to predict therapeutic responses, as well as their potential therapeutic applications against antitumor drug resistance.

**Table 1:** Mechanisms underlying breast cancer drug resistance mediated by exosomes.

Cell origin of exosome	Exosome content	Target(s)	Resistant type	Mechanism	Reference
DOX‐resistant MCF-7 and MDA-MB-231 cells	lncRNA-H19	Unknown	DOX resistance	Promoting cell proliferation and drug resistance, inhibiting cell apoptosis	^25^
Cisplatin-resistant MDA-MB-231 cells	miR-423-5p	Unknown	Cisplatin resistance	Promoting the proliferation, metastasis, and invasion of the recipient cells and slowed apoptosis	^26^
MCF-7 and MDA-MB-231 DOX- and PTX-resistant cells, MCF-7 CSCs	miR-155	TGF-β, FOXO-3a, and C/EBP-β mRNA	DOX and PTX resistance	Promoting EMT and CSC phenotypes and contributing to drug resistance	^82^
ADM-resistant MCF-7 cells	GSTP1	Unknown	Anthracycline/taxane-based neoadjuvant chemotherapy	Detoxification of drugs by conjugating them with glutathione to decrease apoptotic rate	^39^
ADM-resistant MCF-7 cells	UCH-L1, P-gp	MAPK/ERK signaling pathway	ADM resistance	Overexpression of UCH-L1 enhanced multidrug resistance in breast cancer	^37^
MDA-MB-231 cells	miR-1246	CCNG2	DOC, EPI, and GEM resistance	Promoting cell proliferation, migration, and drug resistance	^43^
Circulating exosomes in peripheral blood from BC patients	TRPC5	P-gp	Anthracycline/taxane-based chemotherapy	Stimulating P-gp production in the recipient cells through a Ca^2+^- and NFATc3-mediated mechanism	^35^
PTX-treated MDA-MB-231 cells	Survivin	Unknown	PTX resistance	Promoting cell survival and drug resistance	^42^
DOC-resistant variant of MCF-7 cells	P-gp	Promoting drug efflux	DOC resistance	Transferring drug resistance as well as P-gp from drug-resistant BC cells to sensitive ones	^24^
DOX‐resistant MCF-7 cells	DOX	Unknown	DOX resistance	DOX accumulation in shed vesicles	^27^
TAM- and metformin-resistant MCF-7 cells	Unknown	Unknown	TAM and metformin resistance	Decrease in ERα activity and parallel activation of Akt and AP-1, NF-κB, and SNAIL1 transcriptional factors	^50^
Patients with hormonal therapy-resistant metastatic BC	mtDNA	Unknown	Endocrine therapy resistance	Promoting ER-independent oxidative phosphorylation	^55^
ER^+^ BC cells	lncRNA-UCA1	Cleaved Caspase 3	TAM resistance	Decrease the intracellular level of cleaved Caspase 3; thus impairing tamoxifen‐induced apoptosis	^53^
TAM-resistant BC cells	miR-221/222	P27 and ERα	TAM resistance	Increasing cell proliferation by downregulating P27 and ERα protein levels	^54^
Trastuzumab-resistant BC cells	miR-567	ATG5	Trastuzumab resistance	Regulating autophagy	^65^
HER2^+^ SKBR-3 and BT474 cells	lncRNA APAP2-AS1	N/A	Trastuzumab resistance	Unknown	^63^
HER2^+^ BC cells	lncRNA-SNHG14	Bcl-2/BAX signaling	Trastuzumab resistance	lncRNA‑SNHG14 may induce trastuzumab resistance through inhibiting apoptotic proteins and cell apoptosis via Bcl‑2/Bax pathway	^62^
HER2^+^ BC cells	TGF-β1 and PD-L1	Unknown	Trastuzumab resistance	Neuromedin U in HER2-positive BC cells promotes evasion of the immune response, increasing the expression of TGF-β1 and PD-L1 and functionally affecting ADCC	^61^
HER2^+^ SKBR-3 and BT474 cells	HER-2	Unknown	Trastuzumab resistance	Binding directly to the Trastuzumab and block its activity in vitro	^60^
Basal-like breast cancer cells	PD-1	Unknown	Immunosuppression	ESCRT-related protein ALIX regulated EGFR activity and PD-L1 surface presentation in BC cells	^69^
Highly metastatic BC cells	Unknown	Unknown	Immunosuppression	Suppressed T cell proliferation and inhibited NK cell cytotoxicity	^70^
MSCs	TGF-β, C1q, and semaphorins	Driving PD-L1 overexpression	Immunosuppression	Inducing differentiation of monocytic myeloid-derived suppressor cells into highly immunosuppressive M2-polarized macrophages at tumor beds	^71^
Mouse mammary tumor TS/A cells	Unknown	Unknown	Immunosuppression	Inhibit IL-2-stimulated NK cell tumor cytotoxicity	^72^
BC cells under hypoxic conditions	Unknown	Unknown	Immunosuppression	Suppressed T cell proliferation via TGF-β	^73^

*ADCC* antibody-dependent cell cytotoxicity, *ADM* adriamycin, *AGAP2-AS1* AGAP2 antisense RNA 1, *AP-1* activator protein-1, *ATG5* autophagy-related 5, *BAX* Bcl-2-associated X, *BC* breast cancer, *Bcl-2* B cell leukemia/lymphoma 2, *CCNG2* Cyclin-G2, *C/EBP-β* CCAAT-enhancer-binding protein-beta, *CDK6* cyclin-dependent kinase 6, *CSC* cancer stem cell, *DOC* docetaxel, *DOX* doxorubicin, *EGFR* epidermal growth factor receptor, *EMT* epithelial–mesenchymal transition, *EPI* epirubicin, *ER* estrogen receptor, *ERK* extracellular-regulated protein kinase, *ESCRT* endosomal sorting complex required for transport, *GEM* gemcitabine, *GSTP1* glutathione *S*-transferase P1, *HER-2* human epidermal growth factor receptor 2, *IL-2* interleukin-2, *M2* type 2 macrophages, *MSC* mesenchymal stem cell, *mtDNA* mitochondrial DNA, *NF-κB* nuclear factor Kappab, *NK* natural killer, *PD-L1* programmed death ligand 1, *P-gp* permeability glycoprotein, *PTX* paclitaxel, *TGF-β* transforming growth factor beta, *UCH-L1* ubiquitin carboxyl-terminal hydrolase-L1, *MAPK* mitogen-activated protein kinase, *TRPC5* transient receptor potential channel 5, *NFATc3* nuclear factor of activated T cells, cytoplasmic, calcineurin-dependent 3, *SNHG14* small nucleolar RNA host gene 14, *TAM* tamoxifen, *UCA1* urothelial cancer associated-1.

## Exosome and chemoresistance in BC

**Fig. 1:**
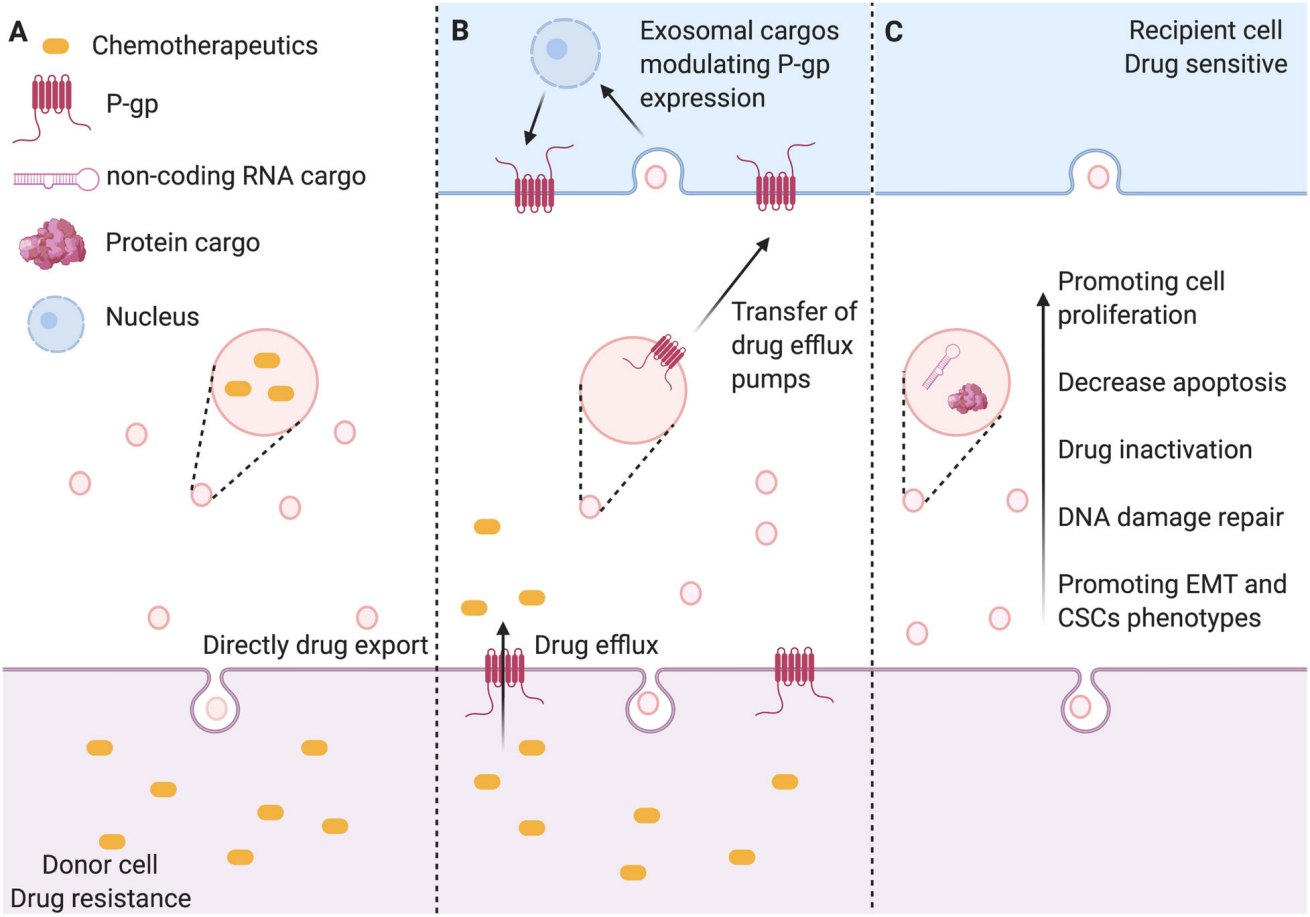
Exosome-mediated mechanisms underlying BC chemoresistance. **A** Chemotherapeutic drugs are secreted when they are encapsulated in the exosomes. **B** Exosomes mediate horizontal transfer of membrane-embedded drug efflux pumps to sensitive cancer cells to favor the drug efflux. Exosomes also deliver functional proteins/miRNAs to upregulate P-gp expression in sensitive cancer cells. **C** Exosomes transfer bioactive cargos that promote cancer cell proliferation, survival, drug inactivation, DNA damage repair, EMT, and stem-like property. Created with BioRender.com.

Chemotherapy is a standard regime for invasive BC, especially triple-negative breast cancer (TNBC). However, BC cells can employ a variety of mechanisms to escape cell death induced by chemotherapeutic drugs. These mechanisms mainly include the efflux and inactivation of drugs^20^, activation of bypass signaling or pro-survival pathways, enhancement of DNA damage repair^21^, and induction of epithelial–mesenchymal transition (EMT)^22^ and stem-like property^23^. Also, a large body of evidence demonstrated that the content of exosomes secreted by tumor cells changes in response to cellular stress induced by anticancer therapy, resulting in the transfer of drug-resistant phenotypes among tumor cells in BC^24–26^. The exosome-mediated transfer of chemotherapeutic resistance between resistant and sensitive BC cells is shown in Fig. 1.

### Exosomes-mediated drug efflux and inactivation

The sufficient intracellular accumulation of cytotoxic drugs in cancer cells is the prerequisite for the efficacy; thus increased drug efflux is an important action causing chemoresistance. To date, at least three mechanisms of increased chemotherapeutic drug export are known to be mediated by exosomes. The first is exosome-mediated direct drug efflux (Fig. 1a). As early as 2003, a positive correlation between EV shedding-related gene and drug resistance in cancer cell lines was described^27^. They found that BC cells could export the chemotherapeutic drug doxorubicin (DOX) into the extracellular medium through vesicle formation^27^. The second mechanism is the exosome-mediated horizontal transfer of membrane-embedded drug efflux pumps to sensitive cancer cells (Fig. 1b). Adenosine triphosphate (ATP)-binding cassette (ABC) transporter utilizes ATP to efflux various xenobiotics, including anticancer drugs with different structures and properties^28,29^. The ABC subfamily B member 1 gene encodes for the drug transporter, permeability glycoprotein (P-gp)^30^. There are a large number of studies showing that P-gp can be transferred from drug-resistant tumor cells to sensitive cells through exosomes, leading to acquired drug resistance in vitro and in vivo^31–33^. Similarly, Lv et al.^24^ demonstrated that P-gp was transported via exosomes from docetaxel (DOC)-resistant BC cells to DOC-sensitive cells, leading to an acquired DOC resistance. The third mechanism is the exosome-mediated transfer of functional proteins/miRNAs to modulate P-gp expression (Fig. 1b). Transient receptor potential channels (TRPCs) play a crucial role in the upregulation of P-gp in drug-resistant BC cells^34^. It is implicated in the adriamycin (ADM) resistance mediated by the exosomes from ADM-resistant MCF-7 cells. The internalization of TRPC5-containing exosomes led to Ca^2+^ influx through TRPC5 in sensitive MCF-7 cells, which resulted in the upregulation of P-gp^35,36^. Furthermore, exosomal ubiquitin carboxyl-terminal hydrolase-L1 (UCH-L1) was also found to upregulate P-gp expression by activating the mitogen-activated protein kinase/extracellular-regulated protein kinase signaling pathway. ADM-resistant MCF-7-derived exosomes were enriched with both P-gp and UCH-L1 proteins. Treatment of sensitive MCF-7 cells with the UCH-L1-specific inhibitor LDN-57444 prevented drug resistance induced by the internalization of exosomes from ADM-resistant MCF-7 cells^37^. These results suggest that exosomes play an important role in the chemoresistance of BC by both directly exporting chemotherapeutic drugs and mediating the transfer or regulation of drug efflux pump.

On the other hand, exosomes also transfer drug-metabolizing enzymes to induce drug inactivation (Fig. 1c). Glutathione *S*-transferase P1 (GSTP1), which belongs to the family of phase II drug-metabolizing enzymes, can detoxify a variety of anticancer drugs by conjugating them with glutathione^38^. Yang et al.^39^ found that the expression of GSTP1 was much higher in ADM-resistant cells and their secreted exosomes. Exposure of sensitive cells to these exosomes induced sensitive cells to develop a drug-resistant phenotype.

### Exosome-mediated transfer of pro-survival cargo

In addition to disrupting the intracellular accumulation of drugs, exosomes also transfer functional cargos that activate pro-survival signaling and unchecked cell cycle progression, which is favorable for tumor growth and progression (Fig. 1c). For instance, survivin is a pro-survival protein that exists in exosomes from different cancer types^40,41^. Kreger et al.^42^ reported that paclitaxel (PTX) treatment induced the secretion of survivin-enriched exosomes from MDA-MB-231 cells, which highly promoted the survival of PTX-treated fibroblasts and SK-BR-3 cells. Moreover, different non-coding RNAs are found in exosomes to favor the survival of chemoresistant BC cells during treatment. Exosomes derived from cisplatin-resistant MDA-MB-231 cells are characterized by high expression of miR-423-5p. They transferred cisplatin-resistant phenotypes to recipient cells by promoting the proliferation, metastasis, and anti-apoptotic signaling^26^. Furthermore, Wang et al.^25^ found that a high level of exosomal long non-coding RNA (lncRNA)-H19 caused DOX resistance in BC cells. Inhibition of lncRNA-H19 significantly reduced the resistance to DOX. Similarly, exosomal miR-1246 was found to promote DOC, epirubicin, and gemcitabine resistance by inhibiting Cyclin-G2 in BC cells^43^. Accordingly, these studies suggest that the transfer of functional proteins and non-coding RNA mediated by exosomes is one of the mechanisms of chemoresistance.

To date, several large-scale validation studies were performed to determine the exosomal protein and miRNA expression profiles in BC after chemotherapy^44–48^. These studies would provide a comprehensive understanding of the function of dysregulated exosomal miRNAs and proteins, which might be helpful to understand the transmission of chemoresistance mediated by exosomes and to overcome chemoresistance in BC treatment. Besides, differentially expressed non-coding RNAs and proteins in exosomes from chemoresistant BC cells support their potential use as disease biomarkers to predict chemotherapeutic response in BC patients.

## Exosome and hormonal resistance in BC

Since approximately 70% of BC patients carry tumors with a high level of estrogen receptor-α (ERα), targeting ERα is an effective hormone therapy^49,50^. A variety of ER-blocking drugs are now used to treat ER^+^ BC patients. Tamoxifen (TAM) is such a drug that can competitively block the ER activation and effectively inhibit ER^+^ tumor growth^51^. However, the effectiveness of hormone therapy in BC is often terminated by the progression of tumors that develop an acquired hormonal resistance after long-term treatment^52,53^.

The mechanism of hormonal resistance is well studied. The ERα dysregulation, the imbalance between receptor activators and inhibitors, and the activation of hormone-independent pathways are the major causes of hormonal resistance^50,53–55^. Semina et al.^56^ demonstrated that exosomes from TAM-resistant MCF-7 cells induced horizontal hormone resistance in estrogen-dependent MCF-7 cells and revealed some key proteins involved. They also found that the co-culture of sensitive MCF-7 cells with exosomes from resistant cells for 14 days caused sensitive cells to develop resistance to antiestrogen drugs^54^.

Exosomal non-coding RNAs play a critical role in the hormonal resistance of BC. Exosomal transfer of lncRNA urothelial cancer associated-1 (UCA1) promoted TAM resistance in ER^+^ MCF-7 cells through mammalian target of rapamycin signaling pathway^57^. Furthermore, exosomal miR-222 mediated TAM resistance in ER^+^ BC cells. The expression of miR-222 is upregulated in exosomes secreted from MCF-7 TAM-resistant cells. Given that the downregulation of miR-222 increased the expression of p27 and ERα at both the mRNA and protein levels and restored the sensitivity to TAM, exosomal miR-222 may promote TAM resistance by upregulating p27 and ERα in ER^+^ BC cells^58^. In addition, Sansone et al.^59^ demonstrated that the horizontal transfer of mitochondrial DNA via exosomes promoted treatment-induced metastasis of cancer stem-like cells from dormancy and led to hormonal resistance in BC.

Overall, the acquired hormone resistance of BC cells can be transferred by exosomes mainly through the following mechanisms: exosomal miRNA- and protein-mediated ERα dysregulation and activation of hormone-independent pathways. Since few studies showed that hormonal resistance was transferred between BC cells, further studies are needed to analyze the exosomes secreted by hormonal resistance BC, including the proteome and non-coding RNA profiles, and to determine the key factors for exosome-mediated transferring of the hormone-resistant phenotype.

## Exosome and resistance to human epidermal growth factor receptor 2 (HER2)-targeted therapy in BC

Overexpression of HER2 is associated with poor prognosis of BC^60^. Hence, HER2-targeted therapy has achieved excellent efficacy in the treatment of HER2^+^ BC^61^. Trastuzumab is the first monoclonal HER-2 antibody approved for the treatment of HER2^+^ BC, which significantly improves long-term disease-free survival of BC patients^62^. Although these drugs have a good clinical response in BC patients initially, a majority of patients turn refractory to HER2-targeted drugs as early as 1 year after treatment completion^63^.

**Fig. 2:**
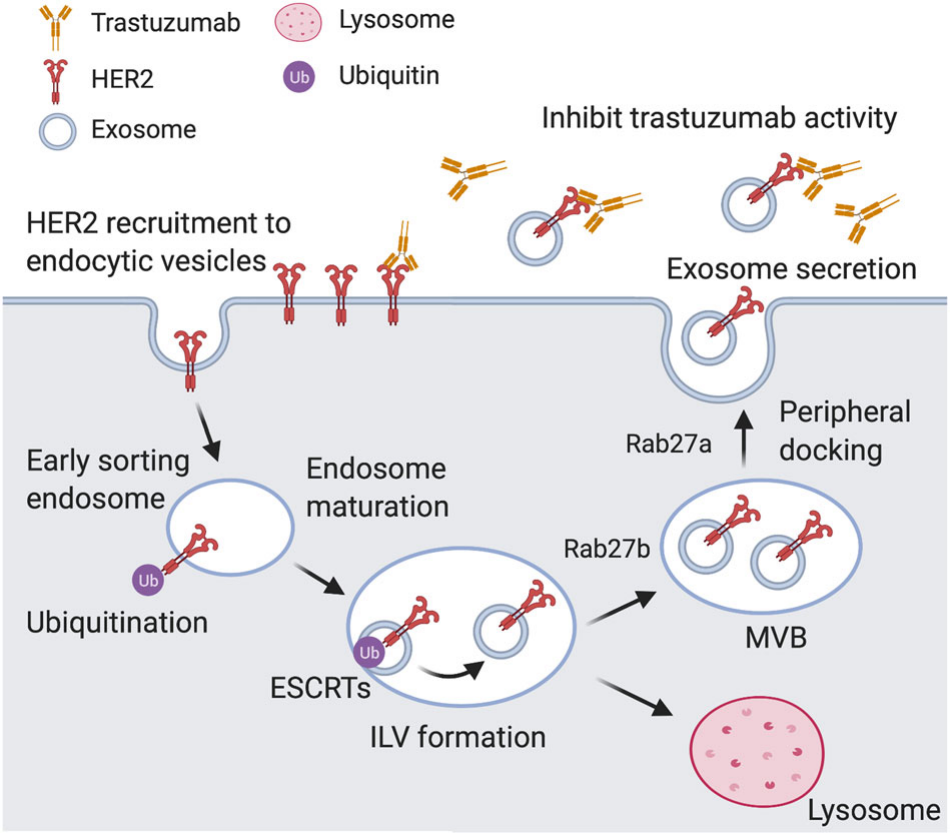
Mechanism of resistance to HER2-targeted therapy and schematic description of the membrane trafficking pathways that underlie multivesicular endosome formation and exosome release. Plasma membrane components are clustered in budding endocytic vesicles that fuse with early endosomes. Early endosome contents are sorted and mature to multivesicular bodies (MVBs) that contain intraluminal vesicles (ILV). During ILV formation, ubiquitinated HER2 are clustered into patches in the membrane by the action of the endosomal sorting complex required for transport (ESCRT) proteins. The majority of MVBs fuse with the lysosomes, resulting in cargo degradation, or release exosomes to the extracellular in a Rab27A/Rab27B-dependent process. Created with BioRender.com.

Some studies have shown that exosome-mediated neutralization of antibody-based drugs is involved in BC trastuzumab resistance (Fig. 2). Exosomes isolated from SK-BR-3 and BT-474 BC cell lines overexpress HER2, which can directly bind to the trastuzumab and block its activity in vitro^64^. Similar results were also observed in patients’ samples.

Martinez et al.^65^ showed that resistance to HER2-targeted drugs was related to the increased levels of transforming growth factor beta 1 (TGF-β1) and programmed death-ligand 1 (PD-L1). They found that EVs transfer these molecules to induce the characteristics of their source cells in drug-sensitive cells. EV-associated TGF-β1 levels are related to the response to HER2-targeted treatment in HER2^+^ BC patients, suggesting that it could be used as a biomarker of therapeutic response.

Dysregulation of non-coding RNAs may also contribute to the trastuzumab resistance. The trastuzumab-resistant BC cells secreted exosomes loaded with the lncRNA-small nucleolar RNA host gene 14 (SNHG14) that induced trastuzumab resistance by inhibiting cell apoptosis via B cell leukemia/lymphoma 2 (BCL-2)/Bcl-2-associated X pathway. Also, the exosomal expression of lncRNA-SNHG14 in serum was highly upregulated in patients who exhibited resistance to trastuzumab compared with patients who responded to trastuzumab^66^. Similarly, the lncRNA AGAP2 antisense RNA 1 (AGAP2-AS1)-containing exosomes also enhanced trastuzumab resistance in BC cells^67^. The heterogeneous nuclear ribonucleoprotein A2B1 (hnRNPA2B1), an RNA-binding protein, was found essential in loading lncRNA AGAP2-AS1 into exosomes^68^. However, the exact mechanism by which exosomal AGAP2-AS1 contributes to trastuzumab resistance needs further investigation. Additionally, by analyzing publicly available miRNA expression profiling data of BC in the Gene Expression Omnibus database, Han et al.^69^ found that the expression of miR-567 was downregulated during trastuzumab resistance. Interestingly, they also found that exosomal miR-567 reversed trastuzumab resistance via inhibiting autophagy-related 5, whereas knockdown of miR-567 induced resistance to trastuzumab treatment.

Accordingly, these findings reveal the unique role of exosomes in promoting resistance to targeted therapies, which results from the direct interaction of HER2-overexpressing exosomes with targeted drugs or from the exosome-mediated transcriptional alternation that promotes cell survival by HER2-independent pathway.

## Exosome and immunotherapeutic resistance in BC

The recent success of novel anticancer immunotherapies has led to a new era of cancer treatment. BCs were traditionally thought to be poorly immunogenic compared to other cancers, such as bladder cancer, melanoma, and lung cancer^70^. However, recent studies demonstrated that the tumors of TNBC are more immunogenic (showing a higher degree of lymphocyte-infiltrating and higher PD-L1 expression) than the other BC subtypes^71^. Currently, several clinical trials are investigating immune checkpoint programmed death 1/PD-L1 inhibitors as monotherapy or in combination with other therapies, such as chemotherapy, radiotherapy, poly adenosine ribose polymerase inhibitors, or angiogenesis inhibitors in patients with metastatic TNBC^72^. Based on the results of the IMpassion 130 study, anti-PD-L1 drug atezolizumab plus nab-paclitaxel received accelerated approval on March 8, 2019 from the Food and Drug Administration for unresectable locally advanced or metastatic TNBC with PD-L1 expression^3^. However, there is still much work needed to optimize immunotherapy strategies in BC, and identification and implementation of novel biomarkers for predicting immunotherapeutic response are urgently required.

Recent evidence suggests that exosomes also play essential roles in remodeling the tumor immune microenvironment. It has been reported that PD-L1 could be packaged into the exosomes of tumor cells, and exosomal PD-L1 enables cancer cells to evade antitumor immunity by inhibiting T cell activation^73^. Also, exosomal PD-L1 appears to be resistant to anti-PD-L1 antibody blockade^74^. However, PD-L1 expression is heterogeneous and dynamic among different BCs. Among different BC subtypes, it has been proved that basal-like BC cells constitutively express the highest levels of PD-L1^75^. Monypenny et al.^76^ showed that the endosomal sorting complex required for transport-related protein ALIX regulated epidermal growth factor receptor activity and PD-L1 surface presentation in BC cells. They found that ALIX-depleted cells exhibited increased surface levels of PD-L1, conferring an enhanced immunosuppressive phenotype on BC cells. Furthermore, Wen et al.^77^ showed that exosomes derived from highly metastatic BC cells directly suppressed T cell proliferation and inhibited natural killer (NK) activity and hence likely suppressed the anticancer immune response in pre-metastatic organs. Zhang et al.^78^ reported that the murine mammary tumor TS/A cell line-derived exosomes inhibited interleukin-2 (IL-2)-stimulated NK cell activity, while the polyphenol derived from the diet, curcumin, reversed the immunosuppressive effects of exosomes on NK cells. In addition, another study demonstrated that exosomes derived from BC cells suppressed T cell proliferation via TGF-β^79^. On the other hand, tumor-associated macrophages are major contributors to malignant progression and resistance to immunotherapy. Biswas et al.^80^ reported that mesenchymal stem cell (MSC)-derived exosomes promoted BC progression by inducing differentiation of monocytic myeloid-derived suppressor cells into highly immunosuppressive type 2 macrophage-polarized macrophages at tumor beds. The findings have significant implications for understanding the underlying mechanisms of immunosuppression in the TME of BC.

In summary, exosomes carry immunosuppressive molecules known to affect immune cell functions in several different ways, which is well studied in different tumors. As most of the immunotherapy research of BC is in the clinical trial stage, the study of exosome-mediated immunosuppression is still at the beginning and needs to be further studied. Exosomes may be used as a predictive biomarker that could potentially serve as a non-invasive strategy to monitor immunotherapy response in tumors.

## Exosomes regulate EMT, cancer stem cell (CSC), and TME in the drug resistance of BC

### Exosome-mediated enhanced EMT and stemness in the drug resistance of BC

Recent studies have shown that the release characteristics of EVs are usually coupled with cellular phenotypic transformation, including EMT^81,82^ and CSC^83,84^. EMT and stemness promote the EV-releasing phenotype of cells, and tumor-derived EVs could in turn initiate EMT and stemness in tumor cells^85^. Thus it is conceivable that the EMT and CSC phenotypes are involved in promoting tumor progression and the acquisition of therapeutic resistance mediated by exosomes.

EMT is a biologic process of loss of epithelial characteristics and the acquisition of mesenchymal phenotype^86^. Cells with EMT undergo a variety of biochemical changes, such as the loss of the tight cell–cell adhesion and the gaining of invasive, migrative, and anti-apoptotic ability. The exosome is an important mediator of EMT that transforms cancer cells into more aggressive phenotypes. Increasing studies indicated that exosomes could deliver pro-EMT factors to recipient cells, thereby facilitating BC progression, including anti-apoptosis, invasion, metastasis, and chemoresistance^87–90^. The miR-155 is an important regulator in EMT and CSCs^91^. Santos et al.^90^ reported that miR-155 upregulation in DOX- and PTX-resistant cells was associated with EMT. The co-culture of DOX- and PTX-sensitive cells with exosomes derived from CSCs or DOX- and PTX-resistant cells resulted in increased levels of miR-155 and induction of chemoresistance. In addition to these effects, EMT is also induced in cells when transfected with miR-155. Qin et al.^89^ reported that TGF-β2 was significantly upregulated in breast milk exosomes during weaning/early involution. Exosomes loaded with a high level of TGF-β2 can lead to EMT in both cancer and benign cells, with the change of cell morphology and actin cytoskeleton, loss of cell–cell junction, increased alpha-smooth muscle actin and vimentin, and decreased E-cadherin.

CSCs are characterized by self-renewal ability and can differentiate into multiple subpopulations of cells within tumors. CSCs not only drive tumor initiation and growth but also mediate tumor metastasis and therapeutic resistance^92^. Shen et al.^93^ showed that treatment with a sublethal dose of chemotherapeutics induced BC cells to secrete EVs, which stimulated a CSC phenotype, and made cancer cells resistant to therapy. Chemotherapy induced BC cells to secrete multiple EV miRNAs, including miR-9-5p, miR-195-5p, and miR-203a-3p, which simultaneously targeted the transcriptional factor one cut homeobox 2 (ONECUT2), leading to the induction of CSC phenotypes and expression of stemness-associated genes, including *NOTCH1*, *SOX9*, *NANOG*, *OCT4*, and *SOX2*. Inhibition of these miRNAs or restoration of ONECUT2 expression abolished the CSC-stimulating effect of EVs from chemotherapy-treated cancer cells.

These findings reveal a critical mechanism of resistance to chemotherapy by which BC cells secrete miRNA-containing EVs to stimulate EMT- and CSC-like features.

### Directional transfer of drug resistance via exosomes between TME and BC cells

**Fig. 3:**
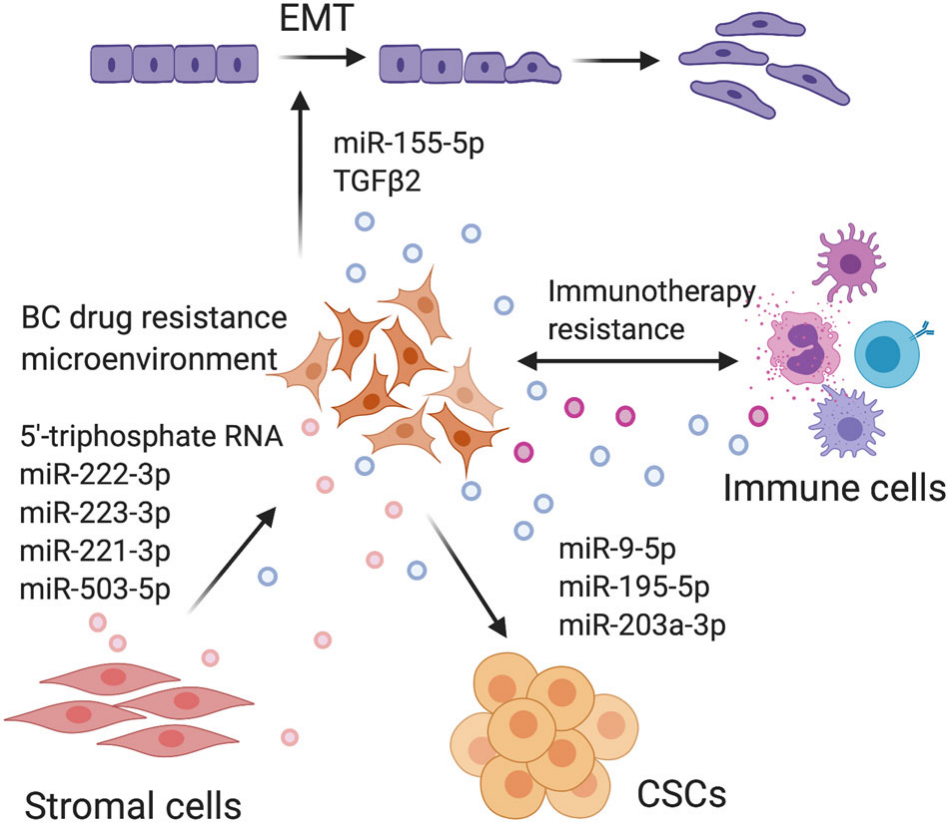
Directional transfer of drug resistance via exosomes between TME and BC cells. Exosomes loaded with a high level of TGF-β2 and miR-155-5p lead to EMT; BC cells secrete miRNA (miR-9-5p, miR-195-5p, miR-203a-3p) containing exosomes to stimulate CSC-like features; exosomes also mediate cellular communication between stromal cells and cancer cells within the TME; stromal exosomes that contained 5′-triphosphate RNA, miR-222-3p, miR-223-3p, miR-221-3p, and miR-503-5p induce resistance in BC cells; exosomes derived from cancer cells, immune cells, and other stromal cells play essential roles in remodeling the tumor immune microenvironment. Created with BioRender.com.

The TME is a heterogeneous population of cells that can be broadly organized into endothelial cells, cancer-associated fibroblasts, and immune cells^94^. Moreover, there are also some non-cellular components, including the extracellular matrix and various soluble cytokines, chemokines, and EVs^95^. It is becoming well established that there are substantial crosstalks between surrounding cells and cancer cells within the TME, promoting tumor development, progression, and drug resistance^96^. TME is characterized with hypoxia, acidic, high oxidative stress^97^. Recent emerging evidence suggests that all these characteristics of TME promote exosome secretion and regulate the content of exosomes by cancer cells, thereby increasing intercellular communication at local and distance sites^98–101^. Here we summarize that, in BC cells, exosomes, as carriers of information, mediate communications between the various cell types in TME leading to the acquisition of drug resistance (Fig. 3).

A variety of examples of exosome transfer between cancer cells and stromal cells that lead to drug resistance have now been reported. Stromal exosomes that contained 5′-triphosphate RNA induced resistance in BC cells through the viral RNA pattern recognition receptor retinoic acid-inducible gene I (RIG­I) and NOTCH3 signaling and activated a signal transducer and activator of transcription 1 (STAT1)-dependent antiviral response^102^. STAT1 is one target of interferon-related DNA damage resistance signature, and it was shown that stromal cells increased radioresistance and chemoresistance by communicating with BC cells in a STAT1-dependent manner^102^. The RAB27B, located at chromosome 18q21.1, is a member of the Rab subfamily of small GTPases^103^. RAB27B promoted the transfer of exosomes secreted from stromal cells to BC cells, then exosomal 5′-triphosphate RNA increased the expression of RIG­I, which enhanced BC radioresistance by inducing interferon-related DNA damage resistance signature expression^102^. The same group reported another study that exosomes derived from stromal fibroblasts contain unshielded RN7SL1 RNA that could also be transferred to immune cells, and the unshielded RN7SL1 drives an inflammatory response by increasing the percentage of myeloid dendritic cell (DC) populations^104^.

In addition, the crosstalk between tumors and the TME also regulates tumor progression and response to anticancer drugs by exosomal miRNAs. MSCs release miR-222/223-containing exosomes that induce quiescence in a proportion of BC cells and induce drug resistance^105^. Increased exosomal miR-503 in plasma was detected in BC patients treated with neoadjuvant chemotherapy, which could be partly due to increased miRNA secretion by endothelial cells. The modulation of miR-503 in BC cells altered their proliferative and invasive capacities. This reveals the involvement of the endothelium in the modulation of tumor progression via the secretion of circulating miR-503 in response to chemotherapy^106^.

TME also contains infiltrated immune cells, such as macrophages, neutrophils, mast cells, myeloid-derived suppressor cells, DCs, and NK cells, and lymphocytes^107^. These cells secrete exosomes to transmit the information of immune activation or immunosuppression, and exosomes carrying tumor-associated antigens interfere with antitumor immunotherapies, which have been discussed in the “Exosome and immunotherapeutic resistance in BC” section.

In summary, at present, the research on exosomes and TME in BC is still limited, and a large number of profiling studies are needed. The evidence we discuss above suggests that exosomes are released by cells within TME in response to antitumor therapies and mediate crosstalk in the TME for the transfer of drug resistance to BC cells. All of these studies indicate that exosomes have a potential role as tumor biomarkers to reflect TME changes and predict therapeutic response.

## Potential clinical applications of exosomes in BC drug resistance

### Exosomes as biomarkers on the prediction of therapeutic response in BC

Biomarkers can be used to predict the response of a tumor to a specific treatment and may reflect tumor sensitivity or drug resistance. Although there has been extensive research on biomarkers in BC for prediction of therapeutic response, few are currently recommended for routine clinical applications^108^. Recently, blood-based monitoring of treatment response and progression via liquid biopsy analysis (circulating tumor cells, cell-free DNA, exosomes, and proteins) is attractive because it is minimally invasive, repeatable, and allows a dynamic assessment of specific molecular markers^109,110^.

With the understanding of exosome biology and its relationship with tumor therapy resistance, the unique features of exosomes (carrying surface markers, their cargos reflect the physiological state of the cell they originated from, relatively stable structure, and presence in almost all biological fluids) make them attractive for the study of early detection of disease progression and as potential biomarkers of response or resistance to therapy^111–113^. Indeed, exosomes have some advantages over other analyses used in liquid biopsies. Exosomes contain molecules from their parental cells and protect their cargos from degradation. Therefore, the analysis of nucleic acid contained in exosomes may be more informative and reproducible than that of vesicle-free circulating tumor RNA or circulating tumor DNA (ctDNA). Second, exosomes are more abundant than cell-free DNA, and they provide the opportunity to get information at the DNA, RNA, and protein level. Combination of the information obtained from exosomes and ctDNA may give more accurate results or information for prediction of therapeutic response in BC and monitoring BC progression.

Several recent studies have investigated exosome-associated biomarkers in patients undergoing chemotherapy. Wang et al.^35^ found that the circulating exosomes carrying TRPC5 were significantly correlated with the expression level of TRPC5 in BC tissues and response to chemotherapy. In addition, increased circulating exosomes carrying TRPC5 after chemotherapy preceded cancer progression and predicted acquired chemotherapy resistance. Therefore, the detection of TRPC5-positive exosomes can be used to monitor chemotherapy resistance in real time. Another group found that the level of serum exosomal lncRNA HOTAIR from BC patients was significantly higher than that in healthy individuals^114^. Importantly, the level of exosomal lncRNA HOTAIR significantly decreased in all patients 3 months after the operation, suggesting that the source of serum HOTAIR is from the tumor tissue, and its level is related to tumor burden and disease invasiveness. Also, a high expression level of serum exosomal HOTAIR was associated with a poorer response to neoadjuvant chemotherapy and TAM therapy^114^. Stevic et al.^115^ showed that some miRNAs were selectively enriched in exosomes of HER2^+^ BC and TNBC and were also related with the clinicopathological parameters and pathological complete response within the BC subtypes. The levels of exosome-associated miR-155 and miR-301 changed significantly after neoadjuvant therapy.

Large-scale validation studies on exosome biomarkers can offer significant insight into tumor therapeutic monitoring. However, there are still some limitations. For instance, a standardized method for collecting, processing, and separation of the exosome sample has not been established. Current separation techniques, such as ultracentrifugation, are time-consuming and cannot achieve high-purity separation. Some alternative methods have been developed based on separation by size, immunoaffinity trapping, and exosome precipitation. However, these methods fail to isolate exosomes exclusively and usually result in complex mixtures of EVs and other components of the extracellular space due to overlapping characteristics^116^. In addition, some microfluidic technologies, such as nano-plasmonic exosome technology, in which the label-free exosome detection is based on the surface plasmon resonance, is not widely used^117^. The process of exosome enrichment is still being optimized, and each process may be optimized for a specific cargo, such as protein, DNA, and RNA^118,119^. Also, the application of exosomes as a biomarker is a challenge, because in circulation tumor-derived exosomes are mixed with exosomes derived from normal cells, which might affect the ability to identify and analyze tumor-derived exosomes. To overcome this, we need to identify tumor-specific exosomes for cancer diagnosis or monitoring. Our laboratory has recently developed a multiplexed assay to detect tumor-specific exosomes for cancer early diagnosis using a panel of existing cancer antibodies (unpublished data). Recently, Hoshino et al.^120^ analyzed 426 human samples by proteomic profiling to identify and characterize tumor-derived EV markers in human tissues and plasma different from normal controls, which could be helpful for tumor detection, determining cancer type, and even tumor therapeutic monitoring.

### Exosomes as novel therapeutic interventions in BC drug resistance

As discussed above, exosomes serve as mediators to promote drug resistance by transferring specific proteins or RNAs. There are several strategies to mitigate the role of exosomes in transferring drug resistance in BC, including blocking exosome secretion from specific cell types, such as stromal and BC cells^121,122^, and inhibiting the incorporation of drug transporter into exosomes that can lead to redistribution and accumulation of drugs in BC cells^123,124^. Furthermore, stimulating the immune response of a cancer patient by removing immunosuppressive exosomes with specific biomarkers from circulation using an extracorporeal filter is another strategy^125^. However, there are still many problems in these therapeutic strategies, such as the lack of specific biomarkers and inhibitors.

Several studies have shown the advantages of the utility of exosomes as naturally derived delivery vehicles for therapeutic agents and vaccines for tumor immune therapy. These advantages include nanoscale size, immune compatibility, low toxicity, prolonged blood circulation time, and relatively stable^126^. As natural carriers, exosomes are safe and effective for targeted drug delivery or therapy. Recently, Li et al.^127^ developed a macrophage-derived exosome-coated poly (lactic-co-glycolic acid) nanoplatform for targeted chemotherapy of TNBC. Similarly, Naseri et al.^128^ exploited the exosomes isolated from bone marrow-derived MSCs to deliver LNA (locked nucleic acid)-modified anti-miR-142-3p oligonucleotides to suppress the expression level of miR-142-3p and miR-150 in 4T1 and TUBO BC cell lines. Aqil et al.^129^ showed that curcumin can be delivered effectively using milk-derived exosomes. Oral administration of exosomal curcumin demonstrated enhanced antiproliferative, anti-inflammatory, and antitumor activities against multiple cancer cell lines including BC compared with the free curcumin. In addition, many peptides, non-coding RNAs, or chemotherapeutic agents can be loaded into exosomes derived from distinct cells using different loading methods and targeted strategies, which makes exosomes as an efficient carrier to enhance antitumor therapy and reverse drug resistance.

It has been shown that tumor-derived exosomes contain and transfer tumor-associated antigens, as well as major histocompatibility complex class I molecules, to DC and consequently induce a T cell-mediated immune response against tumor cells^130^. In addition, DC-derived exosomes have been proven safe for vaccine delivery in multiple phase I trials in different types of cancers^131^. Li et al.^132^ developed a novel HER2-specific exosome-T vaccine using polyclonal CD4^+^ T cells armed with HER2-specific exosomes released from DC and demonstrated its therapeutic effect against HER2^+^ BC in double-transgenic HER2/HLA-A2 mice. This study provides a new therapeutic alternative for trastuzumab-resistant BC patients with HER2-specific self-immune tolerance.

## Conclusions

Although modern antitumor drugs have made significant progress, the occurrence of drug resistance often leads to treatment failure in BC. The exosome is a kind of EVs found in circulation, which has the unique potential to capture the dynamic complexity of cancer and can be used to measure a variety of biological components associated with tumor drug resistance in real time. Despite numerous challenges, exosomes may be used as a candidate biomarker for predicting and monitoring therapeutic efficacy in BC patients and as a potential target or carrier to reverse drug resistance, which occupies an important position in the future of tumor detection, prediction, and treatment.
